# Micro Push-out Bond Strength and Bioactivity Analysis of a Bioceramic Root Canal Sealer 

**DOI:** 10.22037/iej.v12i3.16091

**Published:** 2017

**Authors:** Ceci Nunes Carvalho, Renata Grazziotin-Soares, George Táccio de Miranda Candeiro, Luis Gallego Martinez, Juliana Pereira de Souza, Patrícia Santos Oliveira, José Bauer, Giulio Gavini

**Affiliations:** a *Dental School, CEUMA University, São Luís, Maranhão, Brazil; *; b *Discipline of Endodontics College of Dentistry University of Saskatchewan, SK, Canada; *; c *Dental Sciences Post-graduation Program, Course of Dentistry, Universitary Center Christus, Fortaleza, Brazil; *; d * Center of Materials Science and Technology, Energy and Nuclear Research Institute (IPEN), São Paulo, Brazil; *; e * Discipline of Dental Materials, School of Dentistry, Federal University of Maranhão (UFMA), São Luís, Maranhão, Brazil; *; f *Department of Restorative Dentistry, School of Dentistry, University of São Paulo, São Paulo, Brazil*

**Keywords:** Bioactivity, Bioceramic, Dentine, Micro Push-Out Bond Strength, Root Canal Sealer

## Abstract

**Introduction:**

Bioactive endodontic sealers have been developed to improve the quality of root canal obturation. EndoSequence Bioceramic (BC) Sealer is amongst calcium silicate-based materials recently developed for permanent root canal filling. The objective of this study was to evaluate the bioactivity of BC Sealer and its micro push-out bond strength to dentin compared to AH-Plus (AH) sealer.

**Methods and Materials:**

To perform the micro push-out test, 24 root canals of mandibular premolars were instrumented and divided into two groups (*n*=12). Each root was cut into 4 slices and lumens of the canals were filled with the sealers and submitted to micro push-out test. Failure mode was assessed using scanning electron microscopy (SEM). Bioactivity of BC sealer was investigated with scanning electron microscopy/energy-dispersive X-ray (SEM/EDS) and X-ray diffraction (XRD). Bioactivity assessments were reported descriptively. Bond strength data were analyzed by parametric *t*-test (*α*=5%).

**Results:**

In micro push-out test AH had higher bond strength mean values (16.29 MPa) than BC sealer (9.48 MPa) (*P*<0.05). Both groups had low amount of adhesive failure. SEM showed the presence of a mineral precipitate after 30 days and EDS analysis showed that those precipitates have high proportion of Ca. XRD showed peaks of crystalline phases of calcium carbonate compatible with the bioactivity.

**Conclusion:**

BC sealer showed indications of bioactivity and lower bond strength to dentine compared to AH.

## Introduction

After root canal cleaning and shaping procedures, an effective root filling is required to maintain a microorganisms-free environment and to avoid recontamination of the root canal system [1, 2]. For this, root canal filling materials should closely adapt to the dentin canal walls, aiming to prevent leakage throughout the entire canal and also in the apical region [3-5]. The correlation between the bond strength of filling materials and leakage has been considered as a parameter to assess effectiveness of root canal filling [6-8]. Added to the bond strength to dentin, filling materials that presented a biologic response at the material-dentin interface represent a support to improve the quality of sealing [6, 9].

Bioactive endodontic sealers have been developed to improve the quality of root canal obturation. EndoSequence BC Sealer (BC) (EndoSequence, Brassler, Savannah, GA, USA) is amongst the calcium silicate-based materials recently developed for permanent root canal filling [10, 11]. According to the manufacturer, the components of BC are zirconium oxide, calcium silicates, calcium phosphate monobasic, calcium, hydroxide, filler, and thickening agents. Contemporary studies on BC sealer have documented its several adequate characteristics, including its adhesive property [5, 7, 12, 13].

Investigations on the push-out bond strength of BC sealer have used AH-Plus (AH) as comparison [5, 7, 13, 14]. AH-Plus is considered as a gold standard material in root filling because it has been successfully used for many years [7, 15] and also because its advantages in relation to good adaptation and bond strength in comparison with other materials [4, 16].

As mentioned earlier, the bioactivity is a desirable property for obturation materials. In the 70’s, authors reported that certain compositions of glasses (consisted of SiO_2_, CaO, Na_2_O and P_2_O_5_) were able to bond to the bone tissue [17]. When the glasses were placed in contact with biological fluids, a hydroxyapatite layer connecting the material and the mineral phase of the bone was observed [18]. Besides those previously mentioned bioactive glasses, bioceramic root canal sealers also have shown characteristics that might state some bioactivity property. In 2013, when BC sealer was inserted into root sections and hydrated in the presence of phosphate-buffered solution (PBS), surface precipitates with acicular lath-like morphology were observed. In that case, BC formed a tag-like structure, most likely consisted of either cement itself or crystals. Although BC has produced less apatite crystals and has released less Ca^2+^ ions when compared to Biodentine and ProRoot MTA cements, the formation of a tag-like structure was suggested to be responsible for the BC sealing ability and the bond strength to dentin [9].

The current literature provides several evidences regarding the bond strength of BC sealer to dentin either in favor [7, 12, 14, 19] or against it [5, 13]. Consequently, it is difficult to draw an overall conclusion about the bond strength of BC sealer, because of the distinct methodologies used in studies, such as, the variation in the following topics: instrumentation techniques [7], irrigation solutions [5, 20], obturation techniques [12, 13] presence of smear layer [14] and presence of PBS [19]. In addition, information is limited regarding the bioactivity of BC sealer [9], property closely related to its bond strength. In other words, if the material presented bioactivity, it would improve the bond strength to dentin, due to the developing of a stable bond with dentin by deposition of hydroxyapatite [21].

**Figure 1:**
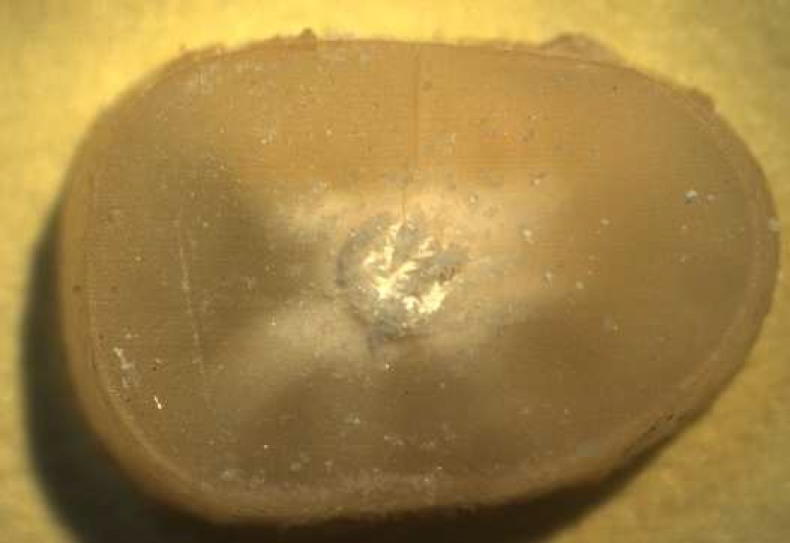
Root dentine slice after immersion in SBF for 30 days, with formation of precipitates on the surface of the endodontic

Therefore, the aims of this study were: *i)* to investigate the occurrence of BC sealer bioactivity by x-ray diffraction analysis (XRD) and to demonstrate it by scanning electron microscopic/energy-dispersive X-ray analysis (SEM/EDS) and *ii)* to assess the bond strength of BC sealer to dentin by micro push-out test using AH-Plus as comparison. The null hypothesis tested was that there was no significant differences in the bond strength of the two tested sealers. 

## Materials and Methods

This *in vitro *study was approved by the Research Ethics Committee from the university where the study was conducted (No.: 0123.0.017.000-11).

***Simulated body fluid preparation (SBF)***

The simulated body fluid (SBF) was prepared according to the protocol previously described [22]. During the SBF preparation process, the solution remained colorless and left no deposits in the receptacle. SBF was stored in a plastic container and kept in a refrigerator at 4^º^C.

***Sample preparation***

Twenty-four recently extracted human mandibular premolars were used. Teeth were included if they had straight, intact and completely formed root, as well as, closed apices. After cleaning, the crowns were removed and the root length was standardized at 15 mm. Root canals were prepared with ProTaper System (Dentsply Maillefer, Ballaigues, Switzerland) up to a master apical file size F5, with irrigation using 1% sodium hypochlorite, and subsequent smear layer removal with 5 mL of 17% EDTA for 3 min.

Roots were randomly divided in 2 groups (*n*=12) according to the sealer used: AH-Plus (Dentsply Maillefer, Ballaigues, Switzerland) and EndoSequence BC sealer (EndoSequence, Brassler, Savannah, GA, USA). Each root was cut into slices in a cutting machine (Isomet 100 Precision Saw, Buehler Ltd, Lake Bluff, IL, USA) under constant cooling with water. After disposal of apical and coronal parts, 4 slices of 1.5 mm thickness were obtained from each root. 

Images of both sides (cervical and apical) of the 48 dentine slices were captured with a digital camera (Q-Color 5; Olympus, America Inc., PA, USA) attached to a stereomicroscopic loupe (SZ61; Olympus America Inc., Center Valley, PA, USA), under ×40 magnification. Then, the lumen diameters of both sides of the slices were measured using the Image J software (National Institute of Health, Maryland, US). Subsequently, slices were individually immersed in distillated water, into Eppendorf containers, and incubated at 37^°^C for 48 h. The slices were dried, by using an absorbent paper. The lumen of the slices were filled with one of the sealers and compressed between a glass slide and a polyethylene matrix. The sealers were mixed according to the manufacturer’s instructions. Finally, the specimens were stored in SBF for 30 days at 37^°^C.

***Micro push-out bond strength***

After storage period, the cervical side of each test specimen was placed in contact to a support (Odeme, Joaçaba, SC, Brazil), which was coupled to the base of a universal test machine (Instron, 3342, Canton, MA, USA). Loading was performed at a crosshead speed of 0.5 mm/min until the sealer was dislodged from the root slice. The bond strength of each slice was calculated as the force (N) of failure divided by the bonded cross-sectional surface area and expressed in MPa. The bonded area of each section was calculated using the following formula: *π*(*r*_1_+*r*_2_)×√((*r*_1_–*r*_2_)^2^+*h*^2^) where *π* is the constant 3.14, *r*_1_ and *r*_2_ are the smaller and larger radii respectively and *h* is the height of the section in mm.

***Failure mode analysis***

All specimens were examined with SEM/EDS (Tabletop Microscope TM3030; Hitachi, Tokyo, Japan). The pushed-out specimens were cleaved longitudinally and the root segments were observed without coating under ×80 magnification to measure the percentage of residual filling material. The interface area (sealer/dentin wall) was classified into three failure modes as follows: >75%: cohesive within the sealer, <25%: adhesive, >25% to <75%: mixed [23].

Six specimens from BC sealer that had been previously submitted to micro push-out bond test and failure mode analysis were used. Different areas were selected from each specimen in interface area (sealer/dentin wall). These areas were observed using a SEM/EDS to identify the presence of elements. 

***X-ray diffraction analysis***

Remaining BC specimens from the micro push-out bond test were used. The precipitate formed on the dentin wall surfaces of the slices was obtained by scraping a blade on this surface (Figure 1). The precipitate obtained was analyzed in a x-ray diffraction (Rigaku - Ultima-IV, Cu _K-α_ radiation) to determine the crystalline phases present in the mineral formation on surface of BC sealer. 

***Data analysis***

For the bond strength test, statistical analysis was performed using parametric *t*-test (GraphPad Prism; GraphPad Software, Inc., La Jolla, CA, USA). The level of significance was set at 0.05. The sample unit was the root (*n*=12), which had derived one single value. The failure mode was analyzed descriptively in percentage (%). The XRD analysis, as well as, the micrographs and graphs from SEM/EDS were also analyzed descriptively.

## Results

Results from the micro push-out bond strength test and failure mode analysis are shown in Table 1. Group AH-Plus had higher mean values that were statistically significant from that of BC sealer (*P*<0.05). Both groups had lower amount of adhesive failure.

**Figure 2:**
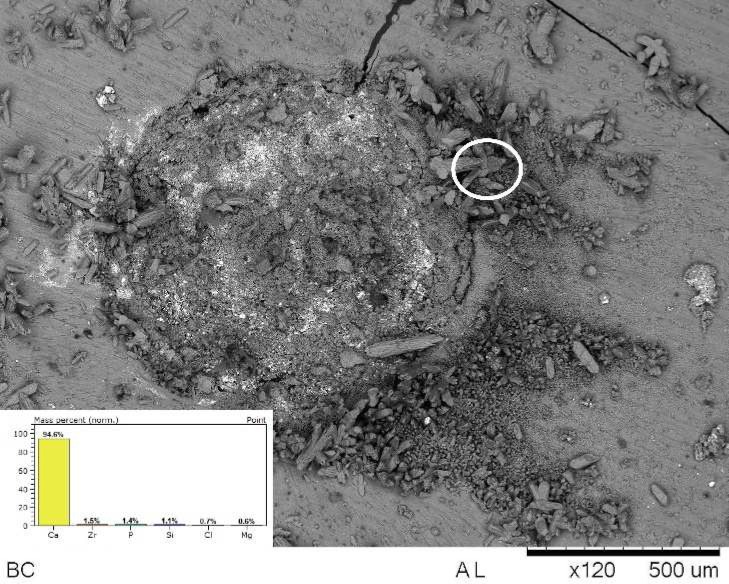
SEM/EDS analysis of interface of Endosequence BC Sealer and root dentine showed a precipitated with chemical elements: Ca, Zr, Mg, Si, P and Cl

Figure 1 shows stereomicroscopic loupe show the presence of precipitate. Figures 2 (on top) and 3 (internal wall) show photomicrographs from SEM/EDS analysis and graph from EDS analysis of BC. Mineral formation composed of Ca, Zr, P, Si, Cl and Mg was observed. Figure 4 shows the diffractogram originated from XRD analysis performed with BC sealer showing peaks of zirconium oxide and calcium carbonate.

## Discussion

A tridimensional filling of the root canal with a connection between filling material and dentin is fundamental to the success of root canal treatment [1, 3, 4], and therefore, every effort to improve clinical results is welcome. The present study was motivated by the relevance of an effective root filling and by the current inexistence of studies that combine different test methodologies to investigate bond strength and bioactivity of EndoSequence BC sealer. Significantly lower bond-strengths were found for BC sealer (9.48 MPa) compared with AH-Plus (16.29 MPa). The null hypothesis was, therefore, rejected.

**Table 1:** Mean (SD) micro push-out bond strength (MPa) of experimental groups,; failure modes are expressed as percent (Different letters indicate the presence of significant statistical difference

**Sealer **	**Mean (SD) Micropush-out bond strength**	**Failure Mode (%)**		
		**Adhesive**	**Cohesive**	**Mixed**
**AH-Plus**	16.29 (2.56)^ a^	6.8	73.55	19.65
**BC Sealer**	9.48 (1.72)^b^	15.5	51.1	32.39

**Figure 3:**
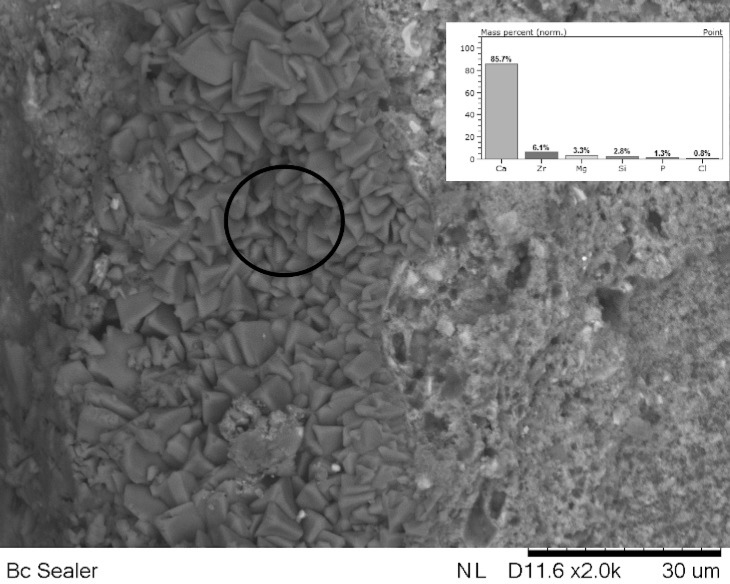
Internal root canal surface impregnated with a precipitate with high calcium concentration

**Figure 4:**
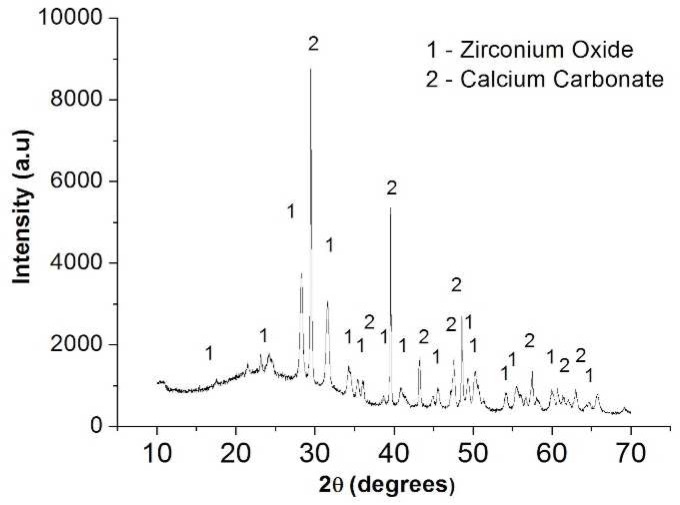
XRD analysis performed on the precipitates formed on the surface of the root dentin slices

Moreover, BC sealer showed high percentage of mixed failure and this can be an indication of its bioactivity (Figures 1 to 4). The lowest bond strength values of BC sealer could be a consequence of using EDTA as irrigating agent, as well as, possible poor hydration process during this experiment. The composition of BC sealer provides the sealer with some chemical bonding ability to mineralized tissues [9]. As EDTA is a chelating agent and removes calcium from the dentin, it might explain why push-out bond strength values were lower. According to the manufacturer, the setting process of BC sealer is dependent on the presence of moisture in the dentinal tubules. In other words, the amount of moisture required for the setting reaction of BC sealer should be provided by the dentinal tubules and it is not necessary to add humidity to the canal prior to the obturation. Although our efforts to provide humidity for the BC sealer specimens during the sample preparation, the insufficient presence of water (SBF was used in this study) might have interfered in hydration, leading to a poor and incomplete setting process. 

In this *in vitro* study, it was extremely difficult, if not impossible, to predict an analogous amount of humidity to reflect the ideal clinical situation as stated by the manufacturer. Hence, additional efforts to maintain the BC Sealer specimens humid should be applied in future tests such as: insertion of the samples in saline solution, distilled water, SBF or PBS throughout the mechanical test.

Still regarding the bond strength, a methodological aspect of this study that needed to be addressed was that the dentin slices were filled only with the sealer.

In this study we did not perform obturation combined with gutta-percha or another core material before obtaining the slices, similar to in many other studies [7, 13]. Although the use of sealer without a core material does not represent the clinical situation, gutta-percha could compromise the authenticity of the test. Gutta-percha has the weakest link of bonding in the filling set; consequently, it detaches easily under load application. This assertion was confirmed by Jainaen *et al.* [24] who showed that push-out bond strengths were significantly higher when canals were filled with sealer alone, than those filled with the main cone and sealer. Those authors asserted that filling root canals with sealer only is a valuable procedure to measure bond strength specifically on the dentin-sealer interface.

In the failure mode analysis, both sealers showed a lower amount of adhesive failures: 6.8% for AH-Plus and 15.5% for BC sealer. These low percentages of adhesive failures may be considered an appropriate characteristic of both sealers. Our findings are in agreement with Shokouhinejad *et al.* [14] who also found the majority of cohesive failure for BC when it was inserted in dentin slices and incubated for 7 days. However, we should be cautious to compare results from failure mode analysis for either BC sealer or AH-Plus. The study by DeLong *et al.* [12] showed that BC used with thermoplastic technique (with single cone or continuous waves) had the majority of mixed failures. AH-Plus also had mixed failures most of the times.

The bioactivity of a sealer showed under the detection of specific peaks in the XRD-diffractogram (Figure 4) and the visualization of a mineral formation [apatite layer] on the material surfaces or in the interfacial layer (Figure 3) at the dentin-material may be showed in SEM [25]. As the bioactivity is not expected for AH-Plus, this current study explored the bioactivity property only for BC sealer. 

SEM micrographs showed the presence of a mineral precipitate after 30 days of incubation/immersion in SBF, which could suggest the bioactivity property. SEM/EDS analysis showed that the precipitates had Ca and P (Figures 2 and 3). In addition, other chemical elements, non-belonging to the hydroxyapatite, were found in the precipitates, such as: Zr, originated from the sealer, and Mg and Cl probably originated from the SBF. These findings suggested the bioactivity property for BC Sealer. To obtain more information about the potential of bioactivity of BC Sealer, the XRD analysis was conducted. It is interesting to highlight that our findings regarding the bioactivity property of BC Sealer are in agreement with two previous studies. Han and Okiji [9] demonstrated that BC Sealer, as well as, WMTA and Biodentine released Ca ions, that formed Ca- and P-rich surface precipitates and caused the uptake of Ca and Si into human root canal dentine, after immersion in PBS up to 90 days, indicating the presence of bioactivity. However, Zhang *et al.* [26] concluded that iRoot SP (also denominated BC Sealer) not only induced expression of mineralized-tissue-associated markers, but it also regulated the messenger RNA expression and mineralization of the MG63 cells. Findings that allow the authors to state that iRoot SP has favorable properties regarding the biologic response of the MG63 cells. 

Taking into consideration those studies previously mentioned; it is possible that, in our study, an alternative growth medium, as well as, diverse experimental times could have led to different results. Consequently, further studies are suggested to investigate deeply the potential bioactivity of BC Sealer, due to the clinical importance of this property for healing of periapical tissues, when the sealer is used to obturate the necrotic teeth with apical periodontitis.

## Conclusion

The bond strenght oF BC Sealer to dentin was lower than AH-Plus and BC sealer showed some kind of bioactivity potential.
